# Data Anomaly Detection for Structural Health Monitoring Based on a Convolutional Neural Network

**DOI:** 10.3390/s23208525

**Published:** 2023-10-17

**Authors:** Soon-Young Kim, Mukhriddin Mukhiddinov

**Affiliations:** 1 Department of Physical Education, Gachon University, Seongnam 13120, Republic of Korea; klpga0166@gachon.ac.kr; 2 Department of Communication and Digital Technologies, University of Management and Future Technologies, Tashkent 100208, Uzbekistan

**Keywords:** CNN, data anomaly detection, time-series classification, structural health monitoring, sensor technology, deep learning

## Abstract

Structural health monitoring (SHM) has been extensively utilized in civil infrastructures for several decades. The status of civil constructions is monitored in real time using a wide variety of sensors; however, determining the true state of a structure can be difficult due to the presence of abnormalities in the acquired data. Extreme weather, faulty sensors, and structural damage are common causes of these abnormalities. For civil structure monitoring to be successful, abnormalities must be detected quickly. In addition, one form of abnormality generally predominates the SHM data, which might be a problem for civil infrastructure data. The current state of anomaly detection is severely hampered by this imbalance. Even cutting-edge damage diagnostic methods are useless without proper data-cleansing processes. In order to solve this problem, this study suggests a hyper-parameter-tuned convolutional neural network (CNN) for multiclass unbalanced anomaly detection. A multiclass time series of anomaly data from a real-world cable-stayed bridge is used to test the 1D CNN model, and the dataset is balanced by supplementing the data as necessary. An overall accuracy of 97.6% was achieved by balancing the database using data augmentation to enlarge the dataset, as shown in the research.

## 1. Introduction

Structural health monitoring (SHM) methodologies are extensively examined within the realms of the civil engineering, mechanical engineering, and aerospace disciplines, with the aim of governing and bolstering strategies pertaining to civil infrastructure. These methodologies achieve this by continuously evaluating the real-time configuration, responses, and burdens experienced by structures. Moreover, they prognosticate, discern, and perceive the imminent behavior of diverse engineering systems. An SHM system typically encompasses an array of data transmission modalities, sensors, a data management repository, instruments for data acquisition, analytical procedures for data, modeling of data, alert mechanisms, a graphical user interface for visualization, condition assessment, performance projection, and another graphical user interface for visualization, in addition to requisite software and an underlying operational platform [1,2,3].

Diverse forms of civil engineering infrastructure currently integrate monitoring systems encompassing a broad spectrum of sensors [4,5,6,7,8,9]. These sensors yield extensive volumes of data pertinent to SHM, constituting a pivotal facet of the discipline. The manipulation and utilization of these data fall under the purview of data science and engineering [10]. Processes involving data acquisition, anomaly analysis, data reconstruction, transmission, retrieval, management, extraction, and modeling all pertain to subdomains within data science and engineering. The scope of SHM applications has transcended the examination of individual infrastructure nodes to encompass entire networks, with future aspirations of extending this coverage to encompass entire urban landscapes. The responsible implementation of SHM technology will entail the accumulation of progressively escalating data quantities. An illustrative instance involves China’s Sutong Bridge SHM system, which, through 785 sensors, generates an annual data volume of 2.5 terabytes. Consequently, an exigent requirement for further research emerges, focusing on the efficacious extraction and utilization of SHM data. This underscores the necessity for devising enhanced methodologies to process said SHM data, drawing inspiration from the algorithms currently deployed for managing voluminous datasets. Nonetheless, scholars encounter challenges, given the intrinsic anomalies typically present in SHM data, originating from environmental influences, system glitches, sensor malfunctions, and related issues. A substantial proportion of these anomalies in SHM systems directly stem from inherent sensor limitations and the inherent imperfections associated with data transmission. The amalgamation of such outliers with data pertaining to real-world catastrophes such as maritime collisions, vehicular accidents, or seismic events can undermine the structural analysis and early warning capabilities inherent to SHM systems.

Deviances within the data constitute a conventional origin of challenges encountered by information-intensive systems. These aberrations exert an influence on the evaluation of structural performance, engendering spurious notifications. The process of data preprocessing, alternatively recognized as data cleansing, also engenders considerable expenses owing to its temporal and human resource demands. The imperative requirement materializes for adept algorithms tailored to purify SHM data, thereby instilling enhanced reliability in the context of real-time monitoring and analysis. Given that SHM frameworks hinge upon extant data, any inconsistencies therein possess the potential to yield egregiously inaccurate characterizations of the structural condition. Scholars have shown that anomalies inherent to the foundational measurement data can engender distortions within signal processing endeavors encompassing computations such as power spectral density and erroneous system identification, thereby misleading damage detection outcomes [1]. To illustrate, the incorporation of anomalous measurement data substantially eroded the precision of mode-shape estimations, particularly concerning modes of elevated order [2]. Consequently, to secure precision in the outcomes furnished by SHM, a pragmatic methodology necessitates a formulation that will autonomously detect and classify diverse anomalies.

Numerous research endeavors have been conducted to confront anomalies present in data streams [3]. Conventionally, two avenues rooted in physical principles are employed for the identification and classification of data anomalies: model-based and model-free approaches. Model-based strategies find their underpinning in physical models, which serve as the bedrock for predictive inferences regarding the anticipated sensor measurements. These predictions are subsequently juxtaposed with the raw empirical data, facilitating their evaluation. Sensor output estimations are ascertained via autoregressive modeling techniques, while deviations between the estimated and observed responses are scrutinized to ascertain sensor malfunctions [4,5]. Additionally, propositions have arisen advocating the application of hypothesis tests to unprocessed measurement data, with the intent of establishing conformity with the distribution pattern exhibited by error-free data instances [6,7,8]. In contrast, model-free methodologies typically ascertain data anomalies through proximity-based comparisons of sensor readings, predicated upon the presumption that anomalies manifest shared attributes [9]. Model-based approaches necessitate a distinct model for each plausible sensor aberration, given that most applications are tailored to discern specific deviations. Conversely, model-free approaches frequently grapple with limitations in accuracy, even when confronted with subtle or marginal deviations. Consequently, an exigency exists for the advancement of more refined and scalable modalities for the detection of anomalies within signal streams.

In recent times, the domain of SHM has seen widespread integration and investigation of deep learning methodologies [10,11,12], a trend driven by the rapid advancements within artificial intelligence (AI). The incorporation of deep learning techniques proves pragmatic for capturing the interdependencies between structural responses and assessment indices through a data-centric approach. This is evident in studies focusing on tasks such as binary data anomaly detection utilizing 1D-CNN [13,14], identification of damage based on vibrations [15,16,17], and detection of damage through imagery [18,19,20]. In a study by Ni et al. [13], a pioneering framework for deep learning-enabled data compression and reconstruction was introduced, comprising two distinct phases. Firstly, a 1D CNN was meticulously devised to directly extract salient features from input signals, with a particular focus on the detection of abnormal data, yielding notably high accuracy levels. Subsequently, a novel method for SHM data compression and reconstruction was introduced, employing an autoencoder architecture. This method was tailored to effectively recover data with remarkable precision, even under the constraints of a significantly reduced compression ratio. In order to substantiate the efficacy of their proposed methodology, the researchers utilized acceleration data extracted from an SHM system implemented on a vast long-span bridge. This bridge, a steel box girder suspension bridge located in China, boasts a substantial main span that extends beyond 1000 m. The SHM system itself is outfitted with a comprehensive array of over 170 sensors, encompassing diverse sensor types such as fiber-optic strain sensors, fiber-optic temperature sensors, accelerometers, displacement sensors, and a variety of others. The dataset exclusively encompassed the acceleration data recorded by the suspension bridge’s accelerometers for the specific period from 1 January 2006 to 31 January 2006. Notably, these accelerometers were strategically placed within four distinct sections of the bridge. Zhang et al. [14] introduced a method for identifying the state of structures based on vibration data, employing 1D CNNs. Utilizing the proposed 1D CNNs, the authors established functional relationships between raw vibration data and the states of structures. These 1D CNN models were meticulously designed to detect subtle local structural variations, and their efficacy was confirmed through experimentation on real-world structures. Datasets comprising structural vibration responses were created, incorporating a T-shaped steel beam (in a laboratory setting), a short steel girder bridge (in a testing field), and a long steel girder bridge (in active service). Notably, these three datasets exhibited a progressive increase in data complexity, facilitating a comprehensive evaluation of the 1D CNN method’s performance.

The proliferation of uncertainties profoundly impacting the behavior of civil structures underscores the utility of such approaches. Concurrently, the realm of computer vision (CV) [21,22,23,24,25] is experiencing escalating popularity owing to its potential in addressing intricate challenges, bolstered by the inherent intuitiveness of visual data depiction when juxtaposed with temporal data. CV assumes a pivotal role in anomaly recognition, facilitated by the visual assessment of acceleration data, thereby enabling the identification of diverse forms of anomalous data instances. Consequently, strategies coupling CV and deep learning have emerged to identify anomalies within datasets. The training of CNNs for the classification of data anomalies entails an exploration of the ramifications stemming from both balanced and imbalanced training datasets, encompassing diverse training balance configurations [26,27,28].

In recent times, machine learning has emerged as a prominent and practical approach for real-world SHM applications that rely on vibration data [29]. Based on the nature of the available training data, machine learning can generally be categorized into three classes: supervised, semisupervised, and unsupervised learning. The suitability of these algorithms for SHM applications was succinctly explored by Entezami et al. [30]. Unsupervised learning, in particular, holds significant promise for a wide range of SHM applications, especially in the context of early damage assessment, as it does not necessitate fully labeled data, meaning there is no requirement for prior knowledge about the current or potential damage state of a structure. Anomaly detection stands out as a primary subfield of unsupervised learning, particularly when it comes to feature analysis and classification. Typically, the objective of anomaly detection is to construct a model referred to as an “anomaly detector” that can identify and distinguish anomalies or outliers from normal data samples [31]. Adapting this concept to SHM offers a pragmatic strategy for assessing damage in civil structures. To achieve this, it is sufficient to train an anomaly detector using unlabeled training data, which represent information solely from the undamaged or normal state. Subsequently, anomaly indices or damage indicators are computed for both training and testing data points, and these indices are then compared against a predetermined threshold.

Li et. al. [32] presented a well-structured and technically rigorous method that addresses the issue of random vibrations induced in bridges by vehicle interactions. The study introduced a novel approach, encompassing the derivation of dynamic equations, the construction of a surrogate model using deep neural networks, and the incorporation of Bayesian inference to account for uncertainties. Authors emphasized the practical application of this approach through a case study involving a railway bridge subjected to high-speed train loading, demonstrating its effectiveness and credibility. Additionally, the authors delved into the concept of Bayesian deep learning, emphasizing its significance in quantifying uncertainties within the vehicle–bridge interaction system. Overall, the paper makes a substantial contribution to the field of structural engineering, offering valuable insights into the analysis of vehicle-induced vibrations in bridges.

Bao et al. [33] devised a deep learning-oriented methodology to categorize grayscale images of acceleration data, effecting a transformation of acquired measurements into a suitable format for subsequent classification via deep neural networks. The method adeptly discerns diverse deviations encompassing missing, minor, outlier, square, trend, and drift anomalies, in addition to the more conventional trend and drift irregularities. Its efficacy was evaluated employing an acceleration dataset derived from an expansive cable-stayed bridge, culminating in a notable accuracy of 85.6%. Motivated by this notable achievement, Tang et al. [21] introduced a CNN-grounded technique for detecting data anomalies, prognosticating the same six anomaly categories by employing the grayscale image of acceleration data, coupled with the corresponding FFT spectrum, serving as a dual-channel input to the CNN. Liu et al. [34] and Chou et al. [35] adopted the generative adversarial network (GAN) + CNN and GoogLeNet architectures, respectively, for anomaly identification within picture data derived from acceleration data. These approaches were similarly verified utilizing a one-month subset of data extracted from [21,33]. In a distinct avenue, Mao et al. [36] advanced a GAN framework that harnessed unsupervised strategies, notably autoencoders, to mitigate the temporal and labor-intensive nature associated with label assignment inherent in conventional supervised learning paradigms, thereby uncovering sensor anomalies. The accuracy of the proposed approach was substantiated through the utilization of two datasets originating from a real-world bridge, following the conversion of measurement data into Gramian angular field representations. Across multiple scenarios, data-driven methodologies manifest a remarkable ability to discern data anomalies with precision. While data-driven techniques offer inherent merits, they are not devoid of limitations, including the requisite for extensive training datasets and challenges associated with achieving high precision across all anomaly categories. To this end, an enhanced and autonomous anomaly detection framework can be conceived, harnessing machine learning-based models through a juxtaposition with principles underpinned by physical considerations.

Recently, an encompassing encoder–decoder transformer architecture was harnessed for the prediction of univariate time-series data. Encouraging outcomes have surfaced from this strategy. For instance, Li et al. [37] conducted a comparative assessment, pitting the transformer approach against the conventional statistical technique ARIMA, the contemporary matrix factorization method TRMF, a recurrent neural network-based autoregressive model (DeepAR), and a recurrent neural network-based state space model (DeepState) across four publicly available forecasting datasets. In a parallel way, Lim et al. [38] applied the transformer framework for multihorizon univariate prediction while retaining the exposition of temporal dynamics. Notably, [38] outperformed both conventional and cutting-edge RNN-based imputation algorithms through the employment of an encoder–decoder architecture integrating a distinctive variant of self-attention. This architecture proved effective in the context of imputing missing values within multivariate, geographically tagged time-series data, exhibiting superiority across three publicly accessible datasets and two competitive datasets.

This study endeavors to investigate the utilization of a structural damage assessment technique founded on a 1D CNN architecture. This technique is extended to encompass tasks related to time-series regression and classification, in addition to the unsupervised acquisition of representative features from multivariate time-series data. The scope and aim of damage detection using 1D CNN are highly relevant within the field of SHM, focusing on the assessment of civil structures. This data-driven approach harnesses the power of machine learning and deep learning techniques to effectively monitor structural behavior by analyzing large volumes of time-series sensor data, with the primary objective of enabling early and accurate damage detection. The system’s aim is to provide real-time structural health assessments, automating the analysis process and generating alerts when anomalies or damage are detected, even for subtle issues like “drift” and “outlier”. It also seeks to generalize its effectiveness in various structures and environmental conditions while addressing data imbalances through techniques such as data augmentation.

In contrast to alternative machine learning techniques for damage assessment present within the existing literature, the proposed approach functions directly upon the raw vibration signal, obviating the necessity for preliminary preprocessing or manual feature extraction. The utilization of 1D CNNs merges the processes of feature extraction and classification, resulting in a computationally economical method that lends itself to real-time applications for damage detection. The customary machine learning methodologies for damage detection rely upon manually constructed features, a strategy that not only proves suboptimal but also entails elevated computational intricacies. Conversely, the CNN-based technique introduced here capitalizes on optimally derived features acquired through 1D CNNs, thereby optimizing classification accuracy—an inherent feature that contributes significantly to enhanced classification performance. Furthermore, this study advances upon the CNN-based damage detection method outlined in [16], addressing a primary shortcoming of the aforementioned approach. Specifically, the method explained in [16] necessitates an extensive number of measurement sessions to generate requisite training data, a challenge particularly pronounced in the context of sizable structures. As a response to this challenge, our study introduces a 1D CNN-based methodology that effectively overcomes this limitation, as it only necessitates two sets of measurement data regardless of the dimensions of the monitored structure. Furthermore, in this study, an effort was made to mitigate the issue of data imbalance by applying data augmentation methods with the aim of balancing all patterns.

The subsequent sections of this paper are organized in the ensuing manner: Section 2 undertakes a comprehensive review of prevailing approaches regarding 1D CNN-based techniques for structural damage assessment. Section 3 explains a method for extracting features from time-series data and the proposed 1D CNN model. The experimental results and a thorough discourse on the proposed approach are explained in Section 4. The outcomes of our study and the constraints intrinsic to the proposed technique are expounded upon in Section 5. Lastly, Section 6 encapsulates our work through a succinct summary and outlines prospective directions for future research endeavors.

## 2. Related Works

Recent research has underscored the supremacy of both 1D and 2D convolutional neural networks (CNNs) over traditional methodologies in tackling complex tasks, such as power engine damage detection [39], electrocardiogram signal classification [40,41], and image-based object recognition [42]. Moreover, these research studies, coupled with prior work delineated in [43,44,45,46], highlight the capacity of CNNs to outperform conventional techniques not solely in accuracy but also in computational speed. A pivotal attribute of CNNs is their adaptive configuration, seamlessly fusing feature extraction processes and damage classification procedures into an integrated learning framework. This architecture enables CNNs to simultaneously draw out and acquire the optimal features directly from raw signals. Notably, the authors recently leveraged 1D CNNs to establish a nonparametric approach for structural damage detection [47]. The efficacy of this methodology in discerning and pinpointing structural damage was evaluated within a laboratory environment utilizing a steel grandstand simulator at Qatar University. The methodology was subjected to assessments involving minute damage instances generated by subtly loosening specific bolts at designated connection points between steel beams and girders. A distinct 1D CNN was allocated to each joint within the structure, and each CNN was trained to assess the condition of its respective joint—determining whether the joint remained undamaged or incurred damage—through processing the acceleration signal recorded at the joint. The training process of the CNNs necessitated a substantial number of measurement sessions. In the initial session, acceleration signals from all joints were captured while the structure remained entirely intact. Subsequently, in each subsequent session, acceleration signals from all joints were logged as a single joint was impaired. Given the 30 joints present in the studied steel frame, a cumulative total of 31 measurement sessions were thus conducted to amass the requisite training data. Empirical findings evidenced that the CNN-based damage detection algorithm effectively identified the impaired joint(s) with precision in real-time scenarios.

The limitation observed in the CNN-based approach proposed in [16] pertains to the substantial number of measurement sessions required for the generation of the requisite training data. While this undertaking remains tenable for comparably modest structures characterized by a confined number of joints or vulnerabilities, its feasibility diminishes within the context of expansive civil structures. The replication of the measurement protocol across all potential damage locations within these larger structures proves impractical. In response to this constraint, this study introduces a systematic methodology presenting an alternate nonparametric damage detection technique that harnesses 1D CNNs in an adapted manner. The novel approach circumvents this obstacle by necessitating a mere two measurement sessions to accrue training data, an advantageous trait that transcends the dimensions of the monitored structure. The resultant output assumes the form of a singular score, encapsulating information concerning the overarching structural health of the under-surveillance edifice. The algorithm’s effectiveness is evaluated through the utilization of recorded data extracted from the publicly accessible benchmark study, denoted as the “Experimental Phase II of the Structural Health Monitoring Benchmark Problem”, a compilation established by the IASC-ASCE Structural Health Monitoring Task Group and disseminated in 2003 [48]. The primary objective underpinning the provisioning of this benchmark data was to furnish a unified test platform facilitating the assessment of novel global structural damage detection methodologies.

## 3. Materials and Methods

### 3.1. Overview of the CNNs

**Figure 1:**
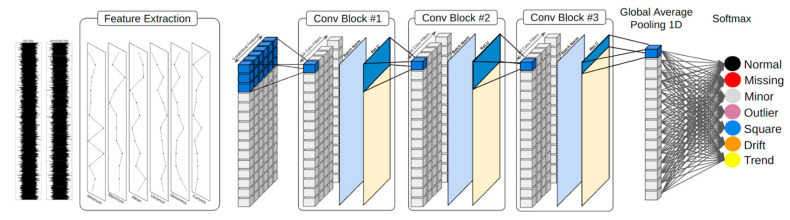
The framework of the proposed model.

A comprehensive visual representation of the systematic methodology that forms the foundation of our study is provided in Figure 1. The procedure is inaugurated with the extraction of six pivotal statistical attributes from the sensor data. Subsequent to this extraction phase, these attributes are channeled as inputs to our customized 1D-CNN model. This model’s architectural blueprint comprises three distinct convolutional blocks, each meticulously designed to capture intricate patterns latent within the statistical attributes. These blocks incorporate Conv1D layers, configured with 64 filters employing a kernel size of 3, accompanied by “same” padding and a stride of 1. Batch normalization layers are interwoven within these blocks, synergistically complemented by the ReLU activation function to introduce heightened nonlinearity. The processed data traverse these convolutional blocks, converging at the global average pooling 1D (GAP1D) layer, which streamlines the spatial information while adeptly preserving the most pertinent feature representations. This course culminates with the application of the softmax activation function, orchestrating the computation of a probability distribution across seven preestablished classes. This final classification assigns the data to the category associated with the highest probability estimate.

In the forthcoming sections, we will undertake an in-depth exploration of each constituent element encapsulated within the schematic diagram delineated in Figure 1. This meticulous examination aims to furnish a more intricate comprehension of our methodological framework, thereby elucidating the underlying logic governing each sequential stage and its consequential import within the comprehensive spectrum of data processing and classification workflows.

### 3.2. Feature Extraction

**Figure 2:**
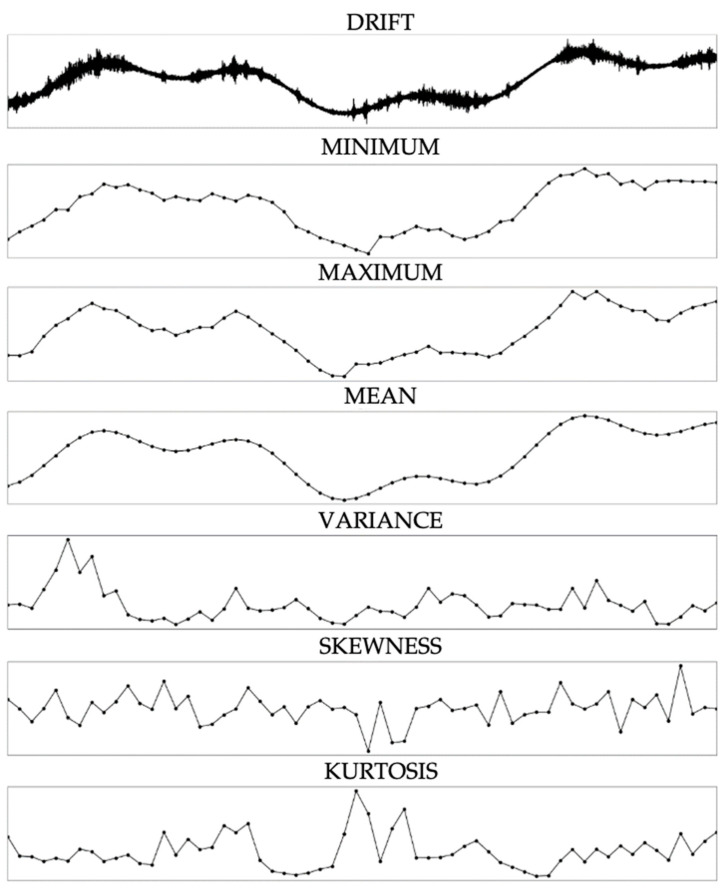
Raw acceleration data for the “drift” class and its corresponding extracted statistical features.

The preliminary stage of our methodological framework revolves around extracting pertinent characteristics from the unprocessed acceleration sensor data. This feature extraction process assumes paramount significance as it condenses extensive data volumes into a discerning ensemble, facilitating enhanced efficacy and expediency during subsequent analysis phases. Presented here is a comprehensive examination of the chosen statistical attributes as shown in Figure 2:

Minimum (Min): This metric encapsulates the minimal observed value within a specific data segment. Its function is to capture the nadir of oscillations or vibrations during the stipulated interval, affording insights into the potential troughs of motion.
Maximum (Max): Unlike the minimum, the maximum denotes the corresponding segment’s pinnacle value within the dataset. This metric illuminates data peaks, which might correspond to heightened stress or perturbation on the bridge structure.
Mean: The mean, emblematic of the arithmetic average across a defined interval, furnishes a central reference point. It indicates the prevailing trend or behavioral pattern encompassing the dataset during the designated timeframe.
Variance: As a pivotal gauge of dispersion, variance furnishes revelations into the degree of divergence exhibited by individual data points with the mean. Elevated variance values may allude to more capricious tendencies or pronounced fluctuations within the acceleration data.
Skewness: This statistical measure scrutinizes the asymmetry or symmetry deficiency within the data distribution. Nonzero skewness values may allude to potential biases or consistent directional tendencies within the data, offering a glimpse into recurrent external influences or patterns impinging upon the bridge dynamics.
Kurtosis: Offering a perspective into the tails of the data distribution, kurtosis appraises the extremeness of data points. Elevated kurtosis values signify a heightened prevalence of outliers or substantial peaks indicative of sporadic external perturbations or anomalous deviations within the bridge’s comportment.

The selection of these specific attributes emanates from their combined capacity to comprehensively encompass the pivotal intricacies characterizing the dynamics of the bridge’s performance. Through the translation of raw sensor measurements into these six defining statistical measures, we acquire a succinct yet potent dataset, fine-tuned to facilitate the ensuing convolutional processing with utmost efficiency.

Figure 2 presents a comparative visualization of the raw acceleration data associated with the “drift” class and its corresponding extracted statistical features, including minimum, maximum, mean, variance, skewness, and kurtosis. The top plot showcases the original sensor readings, illustrating the temporal patterns and changes that characterize the “drift” behavior. The subsequent plots depict the six statistical attributes derived from the raw data. These graphs capture a comprehensive spectrum of the data’s statistical properties, revealing the inherent patterns and providing a quantitative description of the data’s dynamics. By comparing the raw data with its feature representations, the figure underscores the effectiveness of the feature extraction process in translating complex time-series data into more interpretable and manageable formats for further analysis.

### 3.3. D-CNN-Based Model Architecture

Deep learning architectures, with a pronounced emphasis on CNNs, showcase remarkable proficiency in manipulating and categorizing structured data sequences. Within the purview of our investigation, which revolves around time-series sensor data, the employment of a 1D-CNN holds specific relevance. This architectural framework is intricately engineered to discern and internalize localized patterns inherent within sequences, rendering it exceptionally adept for endeavors entailing temporal data analysis. The following is a detailed exposition of our model’s architectural configuration:

Conv1D layer: Each convolutional block initiates with a 1D convolutional layer. We employ 64 filters, each characterized by a kernel size of 3. These filters function as discerners of patterns, gliding across the input sequence to discern localized patterns or configurations. Through the application of “same” padding, the output ensuing from this layer maintains a length equitably matched with the input, thereby preserving temporal information integrity. With a stride of 1, every filter meticulously scrutinizes all conceivable positions within the input sequence, engendering a comprehensive scan.
Batch normalization layer: Subsequent to the convolutional operations, the dataset undergoes normalization within every minibatch. This normalization procedure stabilizes and hastens the learning trajectory. By nurturing a uniform data distribution within each batch, the training regimen acquires heightened resilience against potential fluctuations in feature values, culminating in swifter convergence and, frequently, enhanced generalization capabilities.
ReLU activation function: Following normalization, a rectified linear unit (ReLU) activation function is enacted. This nonlinear function is entrusted with infusing nonlinearity into the model. The selection of ReLU derives from its computational efficiency and the ability to mitigate the challenge of vanishing gradients, thereby fostering more efficacious training for deep networks.
Global average pooling 1D (GAP1D) layer: Following the sequence of convolutional blocks, the architecture of the model accommodates a GAP1D layer. In contrast to conventional pooling layers that curtail dimensionality by opting for maximum or average values within segments, the GAP1D layer averages the responses of all 64 filters across the entire temporal expanse. This begets an unchanging output dimensionality, succinctly capturing quintessential feature information while discarding extraneous or relatively less informative data.
Softmax activation function: The conclusive layer within our architectural construct manifests as a fully connected layer endowed with softmax activation. Given the multiclass disposition of our problem—entailing the possibility of seven distinct classes—the softmax activation engenders the computation of the probability distribution across these classes. Each input is definitively aligned with the class characterized by the highest probability, thereby ensuring a lucid and categorical output.

The design character behind our 1D-CNN model hinges on effectively capturing both local patterns and global characteristics in the sensor data. Our architecture is fine-tuned with convolutional layers, normalization, pooling, and activation functions to provide an accurate and robust classification of the bridge’s behavior.

The structural framework explained in this manuscript comprises two principal components. The convolutional strata synergistically execute both 1D convolution and pooling operations in tandem, thereby affecting the extraction of salient features. Sequentially succeeding these convolutional tiers are fully connected strata, operating as a multilayer perceptron, which assume the role of executing classification endeavors.

The forward propagation is computed in each layer as follows:

(1) $$x_{k}^{l}=b_{k}^{l}+\sum_{i=1}^{N_{l-1}}conv\left(w_{ik}^{l-1},\;s_{i}^{l-1}\right)$$

In the given Equation (1), let $x_{k}^{l}$ represent the input, $b_{k}^{l}$ denote the bias affiliated with the kth neuron at layer *l*, $s_{i}^{l-1}$ signify the output stemming from the ith neuron at the preceding layer, *l* − 1, and $w_{ik}^{l-1}$ represent the kernel spanning from the ith neuron at layer *l* − 1 to the kth neuron at layer *l*. The operation *conv* signifies convolution, wherein the overlapping values of the kernel and the time series undergo multiplication at each position of the kernel, followed by a summation of the resultant outcomes. The activation function $f\left(\;.\right)$ can be judiciously employed with the input $x_{k}^{l}$ to yield the intermediate output $y_{k}^{l}=f\left(x_{k}^{l}\right)$.

For the purpose of training the 1D CNN model, the backpropagation algorithm will be employed to compute the gradient of the loss function $E\left(y\right)$ in relation to the network’s weights. The computation of the derivative of the error concerning each weight is accomplished through the following expression:

(2) $$\frac{\partial E}{\partial w_{i,k}^{l}}=\Delta w_{i,k}^{l}$$

Utilizing the chain rule, it is possible to systematically calculate the gradient on a layer-by-layer basis, commencing the iteration in a reverse sequence from the ultimate layer. The following technique subsequently performs the weight adjustments:

(3) $$w_{i,k}^{l*}=w_{i,k}^{l}+\mu w_{i,k}^{l}$$

Here, $w_{i,k}^{l*}$ represents the weight at the subsequent iteration, and *µ* denotes the learning rate. A comprehensive exposition of the algorithm’s complexities can be found in reference [47].

## 4. Experimental Results

### 4.1. Data Collection and Preprocessing

**Figure 3:**
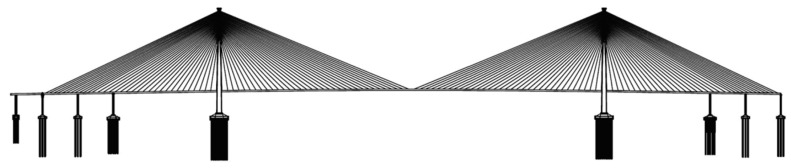
The side view of the cable-stayed bridge in China.

The focal subject of this research encompasses a long-span cable-stayed bridge in China. Illustrated in Figure 3 is the side-view depiction of the bridge, portraying its structural configuration. The bridge features a principal span of 1088 m in length, accompanied by two auxiliary side spans each measuring 300 m. To facilitate the monitoring of its operational condition and behavior, an advanced SHM system was strategically integrated into the bridge. The SHM system is equipped with a network of 16 accelerometers that continually record the vibrational patterns exhibited by the bridge. The spatial arrangement of these accelerometers entails the positioning of 14 dual-axis accelerometers on the bridge deck, with two additional dual-axis accelerometers affixed atop the two towers.

**Figure 4:**
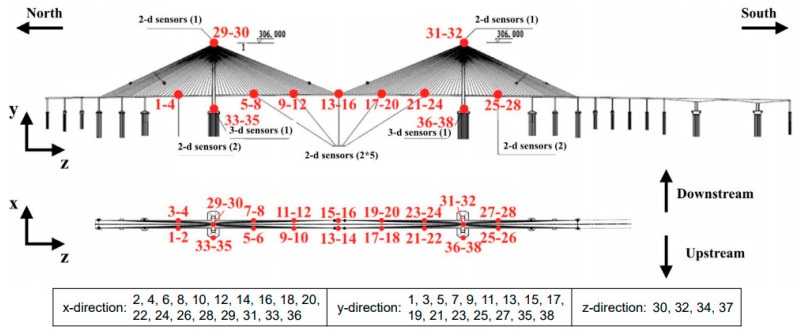
Detailed location of sensors on the cable-stayed bridge [49].

Additionally, two tri-axis accelerometers are positioned at the base of the towers, as visually depicted in Figure 4. This deployment strategy results in the collection of data from a total of 38 channels, with each channel’s vibration signal being sampled at a frequency of 20 Hz. The comprehensive dataset comprises continuous monitoring data spanning a duration of one year. It is noteworthy that a one-month subset of this dataset has been employed in the context of the 1st International Project Competition for Structural Health Monitoring (IPC-SHM, 2020), as expounded upon in reference [49]. In line with the objectives of this study, the aforementioned one-month subdataset serves as the foundation for training and validating the proposed methodology. Regarding annotation, the acceleration dataset is annotated hourly, resulting in a dataset encompassing 28,272 instances of labeled data (31 days × 24 h × 38 channels). Each datum within this dataset corresponds to a continuous one-hour measurement, comprising a total of 72,000 sample points (3600 s × 20 Hz). The core task of detecting data anomalies is executed in this 1 h single-channel acceleration data collection.

**Figure 5:**
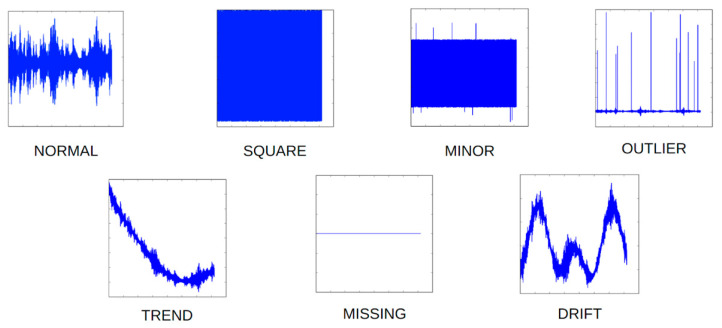
Appearance of each anomaly data category.

The dataset comprises a total of seven distinct classes, denoted as normal, missing, minor, outlier, square, trend, and drift. These classes are assigned labels ranging from 0 to 6, respectively. A comprehensive explanation of each of the seven classes is comprehensively outlined in Table 1. Illustrations depicting representative examples from each category of the dataset are presented in Figure 5.

**Figure 6:**
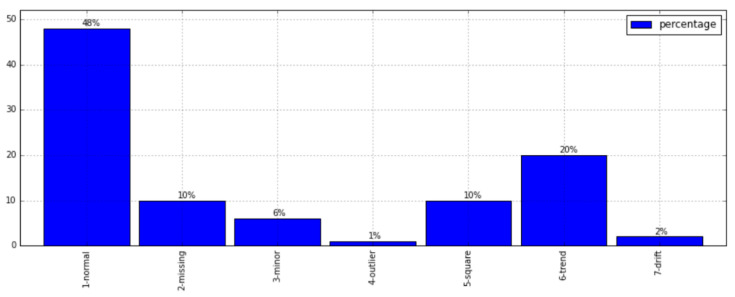
Anomaly data distribution before data augmentation.

Within the dataset, the quantities of data instances within each category are succinctly summarized in Table 2 and Figure 6. Notably, the normal class emerges as the predominant category, while the instances attributed to fault categories remain relatively limited, particularly in the case of the outlier and drift classes. In particular, the data distribution across each category exhibits an inherent imbalance, potentially posing challenges for effectively training a high-performance classifier tailored for data anomaly detection. To address this concern, a data augmentation technique was introduced, specifically targeting the data instances within the fault categories. This augmentation strategy serves to amplify the instances within these categories, thus mitigating the effects of the existing imbalance. 

**Figure 7:**
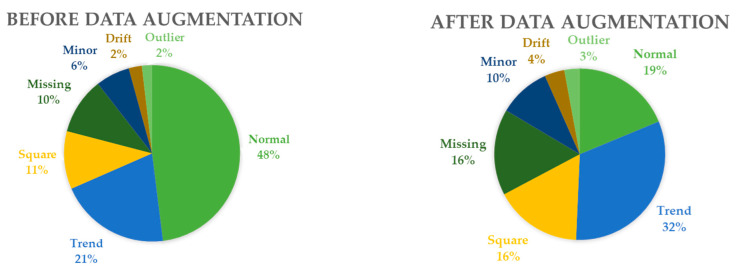
Anomaly data distribution before and after data augmentation.

Importantly, this augmentation process upholds the quantity of training data previously employed in the literature while simultaneously enhancing the scale of the testing data, thereby affording additional validation of the efficacy of the proposed approach. The augmentation procedure encompasses operations such as vertical, diagonal, and horizontal flipping. An illustrative exemplar showcasing the aforementioned augmentation operations is depicted in Figure 7.

As a result, an expanded dataset comprising 72,363 data instances was procured. The augmentative operations demonstrated their efficacy in augmenting the counts of anomalous data instances, thereby mitigating the inherent imbalance within the dataset. A comparison of the proportions of each data class before and after augmentation is depicted in Figure 7.

### 4.2. Evaluation Metrics

The comprehensive evaluation of a classification model’s performance necessitates the utilization of metrics that provide a holistic understanding of its effectiveness across different datasets. Confusion matrixes provide a graphical representation for evaluating the classification efficacy of a model, wherein the predicted labels are compared with the actual labels of a given dataset. This visualization method offers insights into the model’s capacity to accurately classify specific classes, particularly in scenarios involving multiclass classification challenges. In this study, the confusion matrix, accompanied by a comprehensive examination of accuracy, precision, recall, and F1 score, is employed to assess the model’s performance across diverse input datasets [21]. The precise definitions of these metrics are expounded upon as follows:

Accuracy applies to the ratio of correct outcomes, encompassing both true positives and negatives, concerning the overall count of cases subjected to analysis.

(4) $$\mathrm{Accuracy}=\frac{TP+TN}{TP+TN+FP+FN}$$

Precision is the fraction of positive outcomes that accurately correspond to true positive occurrences.

(5) $$\mathrm{Precision}=\frac{TP}{TP+FP}$$

Recall, also known as sensitivity or true positive rate, signifies the ratio of correctly identified actual positive instances to the total number of actual positives in a dataset.

(6) $$\mathrm{Recall}=\frac{TP}{TP+FN}$$

The F1 score, a statistical measure, is computed as the harmonic mean of precision and recall values. It offers a balanced assessment of a model’s performance by considering both the ability to correctly identify positive cases (Precision) and the capability to capture all actual positives (Recall).

(7) $$F1-\mathrm{score}=2\times \frac{\mathrm{Precision}\times \mathrm{Recall}}{\mathrm{Precision}\times \mathrm{Recall}}\times 100$$

In the context of classification evaluation, the terminology is as follows: “True positive” (*TP*) pertains to a sample being accurately categorized into its designated class. “True negative” (*TN*) refers to a sample being correctly recognized as not belonging to a specific class and being correctly placed in another category. “False positive” (*FP*) signifies a sample being incorrectly classified from one of the other categories into the target class. “False negative” (*FN*) designates a sample being misclassified from the target class to one of the other categories.

### 4.3. Results

**Figure 8:**
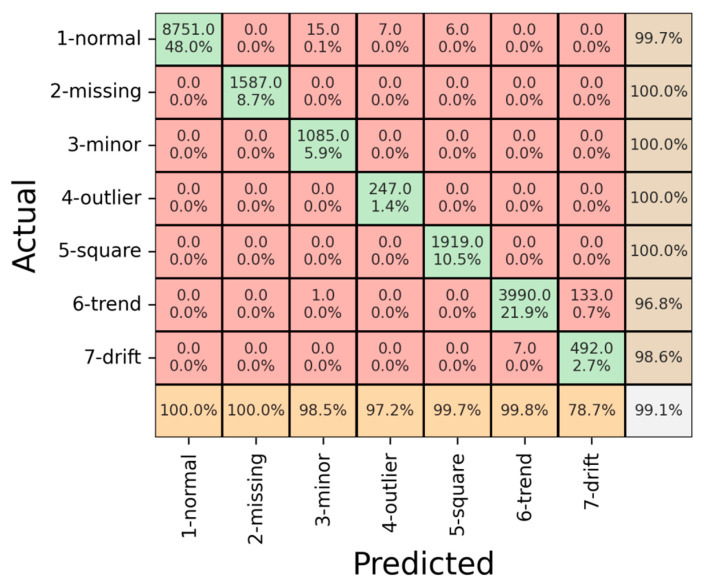
Result of confusion matrix for training data.

The model’s performance was assessed across the above-explained datasets. We divided the dataset into three parts: training, validation, and testing. The outcomes for each part are concisely outlined in Figure 8.

**Figure 9:**
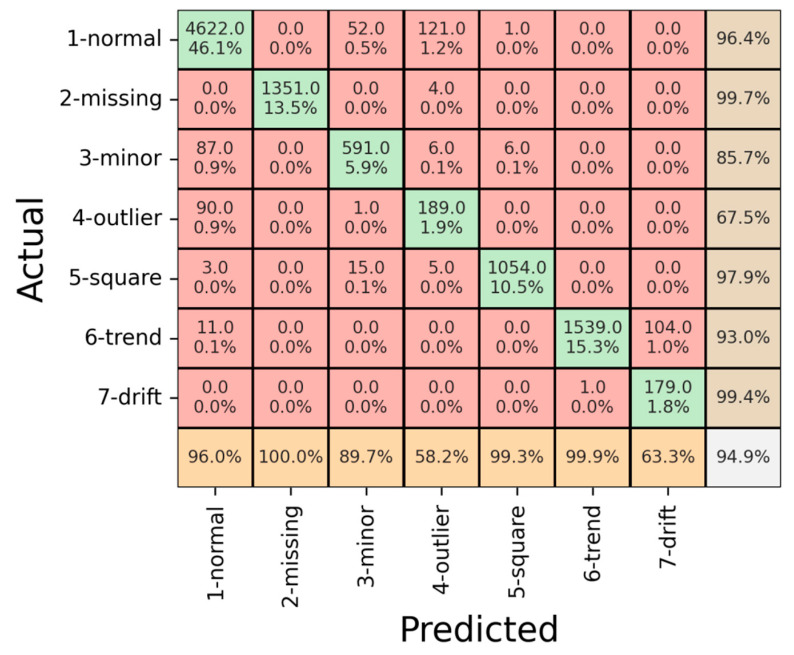
Result of confusion matrix for validation data.

Figure 9 displays the confusion matrix regarding the training dataset. The model exhibited a notable overall accuracy of 99.1%, signifying its adeptness in learning and recognizing the prevalent patterns within the training dataset. Notably, the precision for the “Drift” pattern registered at 78.7%, suggesting occasional instances where the model misclassified other patterns as “Drift”. Conversely, the “Trend” pattern exhibited the lowest recall at 96.8%, indicating situations where the model struggled to identify the presence of the “Trend” pattern correctly. In the remaining categories, the precision demonstrates notable values, particularly in the range of 98.5% and above, including the normal class, and reaching a perfect precision of 100% in the missing class. Correspondingly, the recall metrics exhibit high percentages across the classes, with figures consistently exceeding 98.5%. Remarkably, the missing, minor, outlier, and square classes achieved a perfect recall of 100%.

Subsequently, a quantitative evaluation was conducted using the validation dataset, which offers a comprehensive and informative assessment of the model’s performance as demonstrated in Figure 9.

Figure 9 presents the confusion matrix depicting the model’s performance on the validation dataset. The achieved overall accuracy of 94.9% during validation confirms the model’s respected generalization capability to previously unseen data. Notably, the model exhibited variability in its performance across distinct patterns. Notably, the “Outlier” pattern indicated the lowest precision accuracy at 58.2%, implying occasional misclassifications of actual “Outlier” instances as “Normal”. Similarly, the “Drift” pattern displayed relatively diminished precision at 63.3%, with instances of misclassification of “Drift” patterns as “Trend”. Despite these challenges, the model displayed precision exceeding 89% for the remaining patterns. Regarding recall, the “Outlier” pattern again registered the lowest precision at 67.5%, indicating instances where the model erroneously classified normal patterns as “Outliers”. However, for all other patterns, the recall exceeded 85%. The outcomes of the analysis demonstrate relatively diminished performance in the identification of “drift” and “outlier” patterns. This observation can be attributed primarily to the scarcity of available data instances pertaining to these patterns, as visually depicted in Figure 7. In response to this prevailing data imbalance, we applied data augmentation techniques, resulting in a notable enhancement in classification accuracy. Nevertheless, it is important to emphasize that further bolstering the dataset with an increased representation of “outlier” and “drift” instances could serve to elevate the overall accuracy levels. Furthermore, it is worth noting that the challenges encountered in classification stem from the inherent similarity between “outlier” and “normal” data. In some instances, the model exhibited difficulty in distinguishing between these two classes, leading to occasional misclassifications.

**Figure 10:**
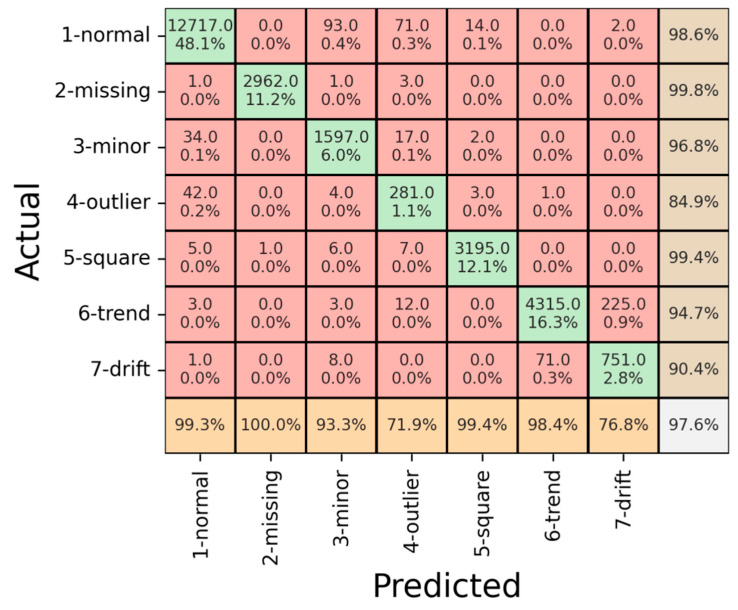
Result of confusion matrix for test data.

Subsequently, a quantitative evaluation was conducted using the test dataset to comprehensively assess the model’s performance as presented in Figure 10. The results of this evaluation offer valuable insights into the model’s effectiveness in real-world scenarios, gauging its ability to generalize and make accurate predictions on unseen data. The utilization of a separate test dataset ensures the assessment’s robustness and validates the model’s reliability beyond the training and validation stages.

As presented in Figure 10, the confusion matrix for the test data offers a comprehensive view of the model’s classification outcomes across various patterns. The achieved overall accuracy of 97.6% on the test data indicates the model’s robustness and proficiency in making predictions on previously unseen instances. This high level of accuracy reinforces the model’s potential for practical implementation in real-world SHM scenarios.

A closer examination of the precision values for different patterns provides valuable insights into the model’s strengths and areas of improvement. Notably, the “Outlier” pattern displayed the lowest precision accuracy of 71.9%, indicating that the model occasionally misclassified genuine “Outliers” as “Normal”. Similarly, the “Drift” pattern exhibited a precision accuracy of 76.8%, whereas some “Drift” patterns were inaccurately identified as “Trend”. These results highlight specific challenges the model encounters in accurately distinguishing certain patterns. Nevertheless, the model showcased robust performance in other classes, achieving precision values exceeding 93%, demonstrating its effectiveness in capturing distinct features of these patterns.

Regarding recall, the model consistently achieved over 84% recall for all patterns, reflecting its ability to identify instances belonging to each category reliably. This consistent recall performance underscores the model’s capacity to generalize and accurately classify diverse structural behaviors. Overall, the quantitative assessment using the test dataset affirms the model’s robustness, underscored by its high overall accuracy and consistent recall rates. The insights gained from analyzing precision values provide valuable directions for refining the model’s performance in challenging scenarios, further enhancing its applicability in real-world structural anomaly detection tasks.

Derived from the confusion matrix, metrics such as precision, recall, and the F1 score play a pivotal role in quantifying the model’s performance by considering various aspects of its predictions. These metrics offer a more nuanced perspective beyond simple accuracy, delving into the model’s ability to correctly identify instances of different classes and its capacity to balance precision and recall.

Table 3 encapsulates the precision, recall, and F1 score for the training, validation, and test datasets, facilitating a comprehensive comparison of the model’s performance across distinct evaluation sets. The precision metric highlights the proportion of true positive predictions among all positive predictions made by the model. In the context of this study, precision aids in understanding how well the model avoids misclassifying instances as belonging to a particular class. Meanwhile, the recall metric measures the proportion of actual positive instances correctly identified by the model, reflecting the model’s ability to capture instances of a specific class. The F1 score, a harmonic mean of precision and recall, provides a balanced metric that considers false positives and false negatives, offering a comprehensive assessment of the model’s overall performance.

**Figure 11:**
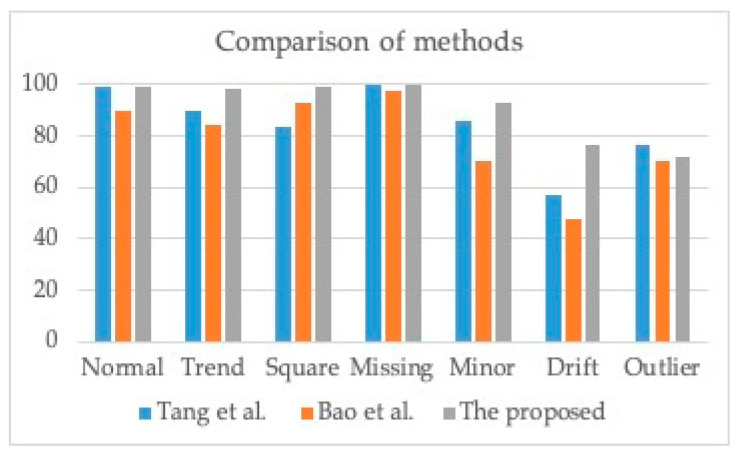
Comparison results between the proposed method and existing data anomaly detectors [21,33].

To provide a comprehensive evaluation of the efficacy of our proposed methodology, we conducted an extensive comparative analysis, contrasting it with various data anomaly detection techniques, including the approaches outlined by Tang et al. [21] and Bao et al. [33]. We further expounded on the performance of our proposed approach and the performance of these data anomaly detectors using a common dataset, as presented in Table 2. It is important to note that while Bang et al. [21] and Bao et al. [33] employed data anomalies spanning a full year, our study focused on publicly available one-month data anomalies. We summarize the outcomes of this thorough comparative analysis in Table 4 and Figure 11, delivering invaluable insights into the effectiveness of our proposed method in the realm of data anomaly detection.

As depicted in Figure 11, our proposed method consistently demonstrates superior performance in most instances. Furthermore, the “outlier” pattern achieves the second-highest result, with a precision of 71.9%, followed closely by Tang et al. [21] with 76.8%. Upon close examination of the comparative results, it is evident that Tang et al. [21] secured the overall second-best performance, while Bao et al. [33] attained the third position in the ranking.

## 5. Discussion

Through a comprehensive combination of experimental investigations and rigorous numerical analyses, the findings of this study substantiate the efficacy and robustness of the proposed approach in the SHM domain. The overarching objective of the approach is to furnish a highly accurate SHM system that possesses the remarkable capability to prognosticate not only the overarching structural condition but also crucial aspects like the size and precise location of any potential damage occurrence.

The proposed methodology introduced in this study presents a novel perspective in the context of structural health monitoring (SHM) data preprocessing, offering a compelling advancement that holds considerable significance for automatic real-time monitoring and the crucial provision of timely alarms within SHM systems. Moreover, it underscores the indispensable role of data-driven offline long-term performance analysis for structures. Notably, while the current investigation is centered on acceleration data, its adaptability extends beyond this specific modality to encompass diverse types of sensor data. This intrinsic flexibility enhances the method’s applicability and opens avenues for integration into a more comprehensive array of SHM scenarios.

While this study sheds light on acceleration data’s potential, future research endeavors should be dedicated to exploring the potency of unsupervised learning techniques tailored to deriving informative representations of anomaly images. Such an undertaking promises to alleviate the need for manual intervention, thereby streamlining and enhancing the efficiency of anomaly detection systems. Furthermore, incorporating a multilabel classification methodology warrants consideration, especially given the multifaceted nature of anomalies that may coexist within the data acquired from SHM systems. This approach’s potential lies in its capacity to concurrently identify and categorize various anomalies within the monitored data, offering a comprehensive and nuanced understanding of structural health.

The proposed approach serves as a catalyst for redefining the landscape of SHM data preprocessing and analysis. As the discipline of SHM continues to evolve, the continued refinement and exploration of this method, coupled with the exploration of emerging avenues like unsupervised learning and multilabel classification, can potentially drive transformative advancements. These advancements, in turn, bolster the efficacy, sophistication, and automation of structural health monitoring systems, thereby contributing to the broader goal of ensuring the durability and reliability of vital infrastructure.

## 6. Conclusions

This study introduces a novel SHM approach, leveraging the power of 1D CNNs for structural anomaly detection. The methodology’s efficacy was substantiated by examining simulated and experimental datasets obtained from a benchmark SHM system. The datasets encompass raw acceleration signals captured from sensors affixed to bridge structures, which underwent progressive damage scenarios under the influence of ambient vibrations. The central principle of the approach lies in harnessing the inherent capability of 1D CNNs to autonomously extract and learn meaningful features from the raw acceleration signals. This process obviates the need for manual feature engineering.

A layer-wise training technique was meticulously employed during the design and training phases to enable the 1D CNN to learn the underlying patterns within the raw acceleration signals proficiently. This strategy allowed for the network’s gradual refinement and the incremental acquisition of specialized features that characterize the structural anomalies of interest. The proposed methodology was subjected to rigorous validation using real-world data from a long-span cable-stayed bridge equipped with an SHM system. This dataset encompassed seven distinct patterns of data anomalies, reflecting various deviations from normal structural behavior.

The experimentation results underscored the proficiency of the designed and trained 1D CNN in detecting data anomalies, yielding an impressive overall accuracy of 97.6% on the test data. Furthermore, the F1 scores, which offer a balanced assessment by considering precision and recall, exhibited strong performance across the seven anomaly patterns at 98.9%, 96.5%, 99.41%, 99.9%, 95%, 83%, and 77.84%, as shown in Table 3. The balance of the training set was identified as a pivotal factor contributing to the system’s overall enhancement. The insights from the data anomaly distribution and sensor-wise anomaly counts provide valuable inputs for subsequent accurate data-cleansing and SHM system maintenance activities.

Comparatively, the proposed deep learning-based 1D CNN approach demonstrated superior efficiency compared to conventional manual inspection methods, suggesting its potential to revolutionize the field of structural anomaly detection within the realm of SHM.

## Figures and Tables

**Table 1:** Detailed clarification of each data class.

Class Name	Description
Normal	The detectors reply in the time domain at a fluctuation angle with no noticeable marks.
Trend	The data have a noticeable gradient (up or down) in the time domain.
Square	The detector’s reply in the time domain is similar to a square wave.
Missing	The detector’s reply in the time domain is missing.
Minor	Compared to the typical sensor response considered normal, the amplitude within the time domain exhibits a minimal magnitude.
Drift	The response of the detector within the time domain is characterized by nonstationarity, leading to unforeseen drift phenomena.
Outlier	The detector’s reply lies outside the range typically observed for regular readings.

**Table 2:** Anomaly data distribution before and after data augmentation.

Class Name	Label	Before Data Augmentation	After Data Augmentation
Normal	0	13,575	13,575
Trend	1	5778	23,112
Square	2	2996	11,984
Missing	3	2942	11,768
Minor	4	1775	7100
Drift	5	679	2716
Outlier	6	527	2108
Total		28,272	72,363

**Table 3:** Results of precision, recall, and F1 score for training, validation and test data.

Class Name	Training Data			Validation Data			Test Data		
	Precision	Recall	F1 Score	Precision	Recall	F1 Score	Precision	Recall	F1 Score
Normal	100%	99.7%	99.8%	96%	96.4%	96.2%	99.3%	98.6%	98.9%
Trend	99.8%	96.8%	98.2%	99.9%	93%	96.3%	98.4%	94.7%	96.5%
Square	99.7%	100%	99.8%	99.3%	97.9%	98.5%	99.4%	99.4%	99.4%
Missing	100%	100%	100%	100%	99.7%	99.8%	100%	99.8%	99.9%
Minor	98.5%	100%	99.2%	89.7%	85.7%	87.6%	93.3%	96.8%	95%
Drift	78.7%	98.6%	87.5%	63.3%	99.4%	77.3%	76.8%	90.4%	83%
Outlier	97.2%	100%	98.5%	58.2%	67.5%	62.5%	71.9%	84.9%	77.8%

**Table 4:** Comparison results between the proposed method and existing data anomaly detectors.

Class Name	Tang et al. [21]	Bao et al. [33]	The Proposed
Normal	98.8	90	99.3
Trend	90.2	84.5	98.4
Square	83.7	93.1	99.4
Missing	99.9	97.7	100
Minor	85.6	70.3	93.3
Drift	57.1	47.9	76.8
Outlier	76.8	70.6	71.9

## Data Availability

The data used in this study are openly available and can be accessed from the following source: [49].
